# Three-stage registration pipeline for dynamic lung field of chest X-ray images based on convolutional neural networks

**DOI:** 10.3389/frai.2025.1466643

**Published:** 2025-03-12

**Authors:** Yingjian Yang, Jie Zheng, Peng Guo, Qi Gao, Yingwei Guo, Ziran Chen, Chengcheng Liu, Tianqi Wu, Zhanglei Ouyang, Huai Chen, Yan Kang

**Affiliations:** ^1^Department of Radiological Research and Development, Shenzhen Lanmage Medical Technology Co., Ltd., Shenzhen, Guangdong, China; ^2^Neusoft Medical System Co., Ltd., Shenyang, Liaoning, China; ^3^School of Electrical and Information Engineering, Northeast Petroleum University, Daqing, China; ^4^College of Medicine and Biological Information Engineering, Northeastern University, Shenyang, China; ^5^School of Life and Health Management, Shenyang City University, Shenyang, China; ^6^Department of Radiology, The Second Affiliated Hospital of Guangzhou Medical University, Guangzhou, China; ^7^College of Health Science and Environmental Engineering, Shenzhen Technology University, Shenzhen, China; ^8^Engineering Research Centre of Medical Imaging and Intelligent Analysis, Ministry of Education, Shenyang, China

**Keywords:** dynamic chest X-ray images, lung field segmentation, medical image registration, anatomical constraints, convolutional neural network, AC-RegNet

## Abstract

**Background:**

The anatomically constrained registration network (AC-RegNet), which yields anatomically plausible results, has emerged as the state-of-the-art registration architecture for chest X-ray (CXR) images. Nevertheless, accurate lung field registration results may be more favored and exciting than the registration results of the entire CXR images and hold promise for dynamic lung field analysis in clinical practice.

**Objective:**

Based on the above, a registration model of the dynamic lung field of CXR images based on AC-RegNet and static CXR images is urgently developed to register these dynamic lung fields for clinical quantitative analysis.

**Methods:**

This paper proposes a fully automatic three-stage registration pipeline for the dynamic lung field of CXR images. First, the dynamic lung field mask images are generated from a pre-trained standard lung field segmentation model with the dynamic CXR images. Then, a lung field abstraction model is designed to generate the dynamic lung field images based on the dynamic lung field mask images and their corresponding CXR images. Finally, we propose a three-step registration training method to train the AC-RegNet, obtaining the registration network of the dynamic lung field images (AC-RegNet_V3).

**Results:**

The proposed AC-RegNet_V3 with the four basic segmentation networks achieve the mean dice similarity coefficient (DSC) of 0.991, 0.993, 0.993, and 0.993, mean Hausdorff distance (HD) of 12.512, 12.813, 12.449, and 13.661, mean average symmetric surface distance (ASSD) of 0.654, 0.550, 0.572, and 0.564, and mean squared distance (MSD) of 559.098, 577.797, 548.189, and 559.652, respectively. Besides, compared to the dynamic CXR images, the mean DSC of these four basic segmentation networks with AC-RegNet has been significantly improved by 7.2, 7.4, 7.4, and 7.4% (*p*-value < 0.0001). Meanwhile, the mean HD has been significantly improved by 8.994, 8.693, 9.057, and 7.845 (*p*-value < 0.0001). Similarly, the mean ASSD has significantly improved by 4.576, 4.680, 4.658, and 4.658 (*p*-value < 0.0001). Last, the mean MSD has significantly improved by 508.936, 519.776, 517.904, and 520.626 (*p*-value < 0.0001).

**Conclusion:**

Our proposed three-stage registration pipeline has demonstrated its effectiveness in dynamic lung field registration. Therefore, it could become a powerful tool for dynamic lung field analysis in clinical practice, such as pulmonary airflow detection and air trapping location.

## 1 Introduction

Compared with computed tomography (CT), magnetic resonance imaging (MRI), positron emission tomography (PET), PET-CT, and other imaging devices, X-ray is the most widely used primary chest imaging technique as it is widely available, low-cost, fast imaging speed, and easy to acquire (Howell, 2016; Seah et al., 2021; Yang et al., 2024). Notably, its characteristic of fast imaging speed (seconds after exposure) makes the X-ray the preferred chest imaging device to improve work efficiency and facilitate the diagnosis of routine, critically ill, and emergency settings in clinical chest imaging examinations (Yang et al., 2024; Yang et al., 2024).

Although traditional static chest X-ray (CXR) images can display gross lesions such as lung inflammation, lung lumps, tuberculosis, etc., they lack corresponding information on dynamic lung motion. Therefore, dynamic CXR images during free breathing are captured to analyze dynamic indicators during lung respiration, such as hemi-diaphragm motion (Yang et al., 2024; Chen et al., 2022) and dynamic cardiothoracic ratio detection (Yang et al., 2024; Jafar et al., 2022). However, for dynamic CXR images, detecting the above dynamic indicators is not enough in clinical practice. It is more important to analyze further the detected dynamic indicators to provide reasonable clinical recommendations. Like the parametric response mapping in expiratory and inspiratory chest CT images (Deng et al., 2024; Wang et al., 2024), registration technology is indispensable to accurately determine the point-to-point relationship between the above dynamic indicators of these dynamic CXR images.

Based on the above, medical image registration technology is a crucial step and pillar problem in medical image analysis for aligning the source image (moving image) with the target image (fixed image) (Mansilla et al., 2020). Essentially, registration technology aims to find the deformation fields of the point-to-point correspondence between the source and target image. Specifically, the classic unsupervised technology for medical image registration, SimpleElastix (Marstal et al., 2016), is widely used in non-deformable tissues such as three-dimensional (3D) brain and two-dimensional (2D)/3D bone X-ray/CT/MRI images (Guo et al., 2022; Guo et al., 2022; Guo et al., 2022; Yang and Guo, 2024; Chang and Lee, 2021). Compared with SimpleElastix, the end-to-end deep convolutional neural network is constantly proposed for the intelligent processing of medical images (Iqbal et al., 2021; Iqbal et al., 2025; Yang et al., 2022). However, it is disappointing that most of the supervised registration techniques for chest medical images are focused on 3D CT images (Xiao et al., 2023), and only a few studies have been conducted on 2D X-ray images from 2016 to the present (Nie et al., 2024). The only registration method, AC-RegNet, for chest CXR images during this period was proposed in 2020 (Mansilla et al., 2020). Specifically, this technique produces anatomically plausible results for 2D chest CXR images. The evaluation metrics of the proposed AC-RegNet surpass the AE-RegNet, CE-RegNet, RegNet, and SimpleElastix (Mansilla et al., 2020; Marstal et al., 2016). Therefore, the AC-RegNet has been considered the state-of-the-art registration architecture for chest X-ray (CXR) images. Nevertheless, the above models have not achieved registration of dynamic lung field images collected during free or forced breathing. Therefore, this limits the quantitative analysis of the dynamic lung field based on the CXR images.

Accurate lung field registration results may be more favored and exciting than the registration results of the entire CXR images for dynamic lung field analysis in clinical practice, such as pulmonary airflow detection (Jiang et al., 2024) and air trapping location (Zhang et al., 2024). Specifically, the lung undergoes a non-rigid and complex process of contraction and expansion during breathing (Santhanam, 2006). Compared to other static organs or tissues (such as the brain, bones, etc.), these non-rigid and complex deformations present significant challenges for lung field registration. Registration is even more challenging for dynamic chest CXR images projected in 2D further. In addition, the limited quantity and quality of dynamic CXR images also constrain the development of dynamic lung field registration. Despite the existing problems and challenges, we still hope to propose a registration model for dynamic lung field images based on the limited quantity and quality of static CXR images to expand the clinical application of dynamic chest CXR images and improve the accuracy of quantitative analysis based on lung fields.

However, the limited number of dynamic CXR images makes it challenging to train the AC-RegNet, which limits the clinical quantitative analysis of dynamic lung fields. Based on the above, we propose a fully automatic registration pipeline for the dynamic lung field of CXR images based on AC-RegNet architecture and static posteroanterior (P-A) CXR images. First, the dynamic lung field mask images are generated from a pre-trained standard lung field segmentation model with the dynamic P-A CXR images. Then, a lung field abstraction model is designed to generate the dynamic lung field images based on the dynamic lung field mask images and their corresponding P-A CXR images. Finally, we propose a three-step registration training method to train the basic architecture (VectorCNN and differentiable warper) of AC-RegNet, obtaining the registration network of the dynamic lung field images. Our contributions in this paper are briefly described as follows:

1. Overall, this paper presents a fully automatic three-stage registration pipeline for the dynamic lung field of CXR images based on AC-RegNet, which effectively addresses the issue of the inability to register dynamic lung field images for the quantitative analysis of lung fields.
2. Essentially, this paper proposes a three-step registration training method that includes initial training, enhanced training, and final training of the basic architecture of AC-RegNet based on static P-A CXR images, which fully utilizes static P-A CXR images to address the registration issue of these non-rigid and complex deformations in the dynamic lung field.
3. Clinically, the proposed fully automatic registration pipeline can effectively register dynamic lung field images to maintain lung field morphology alignment during respiration. Thus, this may become a valuable tool for further quantitative analysis of dynamic lung fields, such as pulmonary airflow detection, air trapping location, etc.

## 2 Materials and methods

### 2.1 Materials

**Figure 1:**
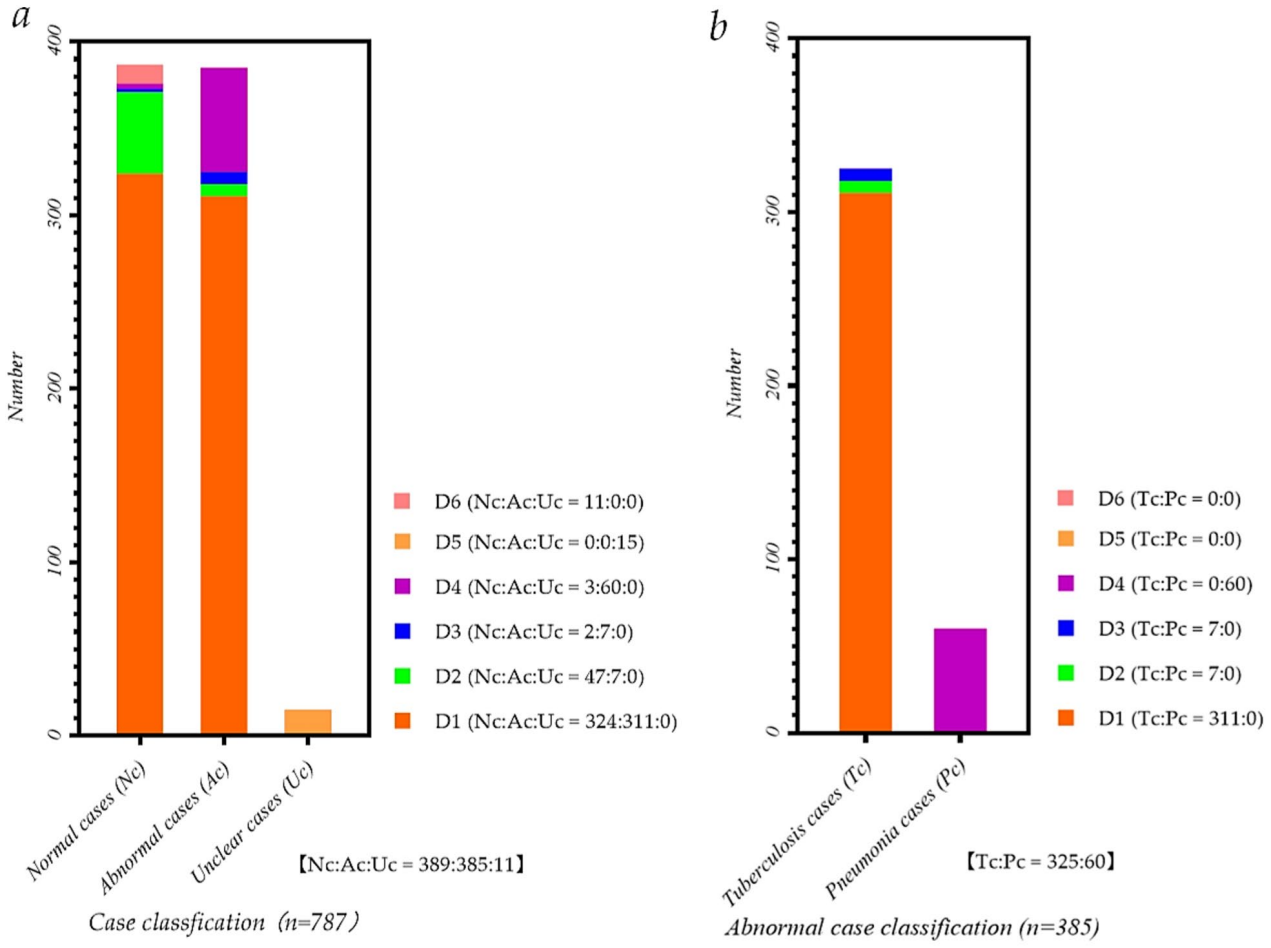
Data distribution map of P-A CXR images in each dataset. **(a)** Case classification map of P-A CXR images in each dataset; **(b)** Abnormal case classification map of P-A CXR images in each dataset.

Figure 1 intuitively shows the detailed distribution of the static and dynamic P-A CXR images in the six datasets. Meanwhile, Figure 1a intuitively shows each dataset’s proportion of normal, abnormal, and unclear cases. Besides, Figure 1b intuitively shows the proportion of pneumonia and tuberculosis cases in each dataset.

Specifically, five datasets, D1-D5, include 786 sets of static 512 × 512 P-A CXR images, and dataset D6 includes 11 sets of dynamic 512 × 512 CXR images. These five datasets, D1-D5, are from publicly available datasets (Yang et al., 2024). Besides, 11 sets of dynamic P-A CXR images of dataset D6 are selected from the case of CXR video. This case of CXR video collected from a female participant aged 53 during free breathing using a digital X-ray imaging system (manufacturer: Lanmage, collection mode: sequence point slice, exposure parameters: 78KV, 200 mA, 50 ms, and flat panel detector: IRAY) for chest photography. Besides, due to the unstable radiation dose during the startup of the digital X-ray imaging system, the 13 video frames and subsequent frames in the video image tend to stabilize. Therefore, these 11 sets of dynamic 3,072 × 3,072 P-A CXR images are 13–23 video frames extracted from the CXR video. Then, to ensure that these dynamic P-A CXR images are the same size as static P-A CXR images, resize them to 512 × 512.

This female participant was provided written informed consent, and the Guangzhou Medical University Ethics Committee in China approved this study (Grant number: 2023-hg-ks-24, Approval Date: 28 August 2023, Tel: +86–20-34153599, and Fax: +86–20-34153066).

### 2.2 Methods

**Figure 2:**
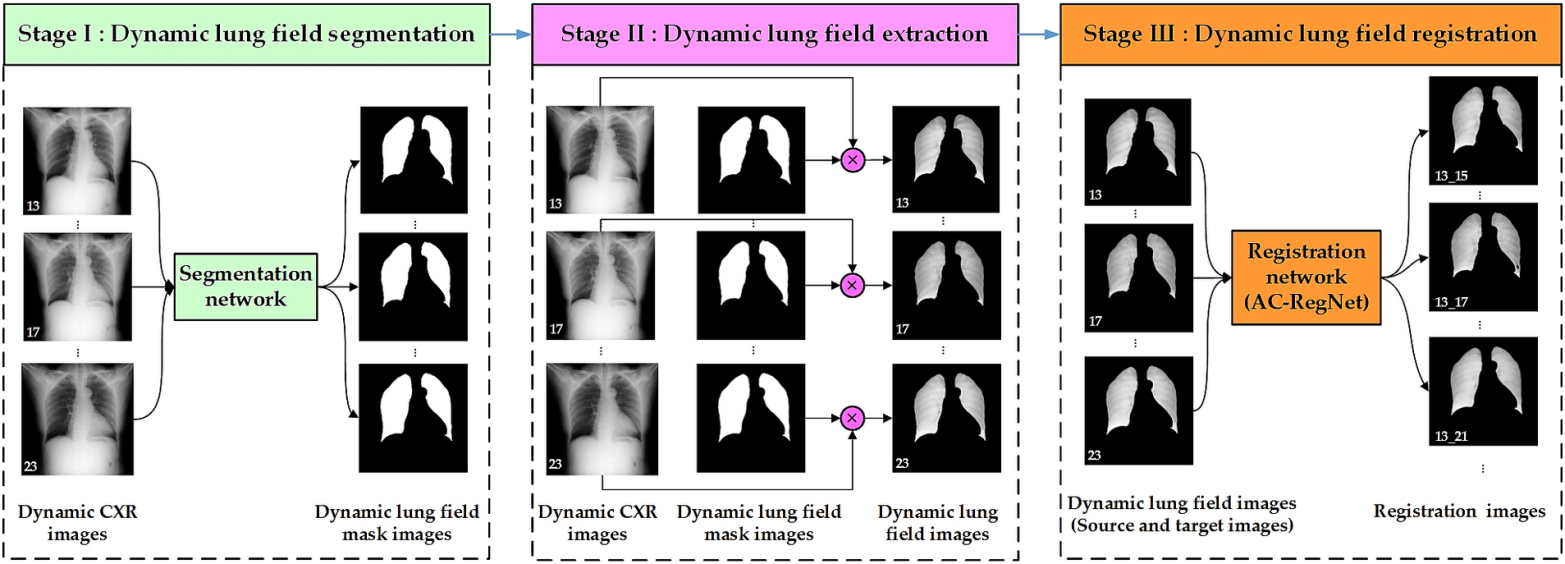
Three-stage structure of dynamic lung field registration pipeline of CXR images based on AC-RegNet architecture.

Figure 2 shows the three-stage structure of the dynamic lung field registration pipeline of CXR images based on AC-RegNet architecture. First, the dynamic lung field mask images of the CXR images are generated by a pre-trained standard and pathological lung field segmentation network. Then, the dynamic lung field mages are abstracted based on the dynamic lung field mask images and their corresponding CXR images. Lastly, dynamic lung field images can be registered at different times based on the pre-trained AC-RegNet (registration network). The source code is available on the website: https://github.com/YingjianYang/Dynamic-Lung-Field-Registration.

#### 2.2.1 Stage І: dynamic lung field segmentation

A cross-center and standard segmentation network of pathological lungs is crucial for extracting lung fields from dynamic P-A CXR images, effectively assisting the registration of dynamic lung field images.

Convolutional neural networks (CNNs) have become the leading technical means for medical image segmentation tasks (Yang et al., 2022; Long et al., 2015; Badrinarayanan et al., 2017; Ronneberger et al., 2015; Yang et al., 2021; Jha et al., 2019; Wang et al., 2021; Zaman et al., 2024; Zeng et al., 2023; Duan et al., 2023), providing excellent technical support for registering dynamic lung fields. Our previous research separately utilized these cross-center 755 static P-A CXR mages and their lung field label images to train five basic CNNs, achieving corresponding cross-center and standard pre-train segmentation networks (Yang et al., 2024). These five CNNs include FCN (Long et al., 2015), SegNet (Badrinarayanan et al., 2017), U-Net (Ronneberger et al., 2015), ResU-Net++ (Yang et al., 2021; Jha et al., 2019), and AttU-Net (Wang et al., 2021). However, the above research found that due to the lack of skip connections between each level in FCN, the pre-train lung field segmentation network based on FCN lacks detailed information and exhibits noticeable jagged edges at the edges of the lung field mask. Therefore, the other four pre-trained lung field segmentation networks can be used to perform lung field segmentation tasks for dynamic P-A CXR images.

#### 2.2.2 Stage ІІ: dynamic lung field extraction

Dynamic lung field images are extracted from the dynamic P-A CXR images to register dynamic lung field images. Like the lung field extraction of the chest CT images for calculating lung parenchyma parameters (Yang et al., 2021), locating chronic obstructive pulmonary disease (Yang et al., 2020), and calculating lung radiomics feature (Yang et al., 2022), the lung field extraction of dynamic P-A CXR images is based on the dynamic lung field mask images generated from the segmentation network and their CXR images.

The pixel values in the lung field of the dynamic lung field mask images should be set to 1 instead of the pixel values in the non-lung field to 0, obtaining the dynamic preprocessed lung field mask images. Then, the dynamic preprocessed lung field mask images are multiplied by their CXR images at the pixel level to obtain the dynamic lung field images. The above pixel values set maintain the grayscale of the dynamic lung field mask images consistent with the grayscale of the lung field in the CXR image.

#### 2.2.3 Stage Ш: dynamic lung field registration

Since the AC-RegNet architecture was proposed, this technique has produced anatomically plausible results and has been considered the state-of-the-art registration architecture for CXR images (Mansilla et al., 2020; Nie et al., 2024). In this work, we extend the application of the AC-RegNet architecture to the registration of dynamic lung field images. Specifically, this basic architecture of AC-RegNet includes two main modules: VectorCNN and differentiable warper (Mansilla et al., 2020; Jaderberg et al., 2015).

**Figure 3:**
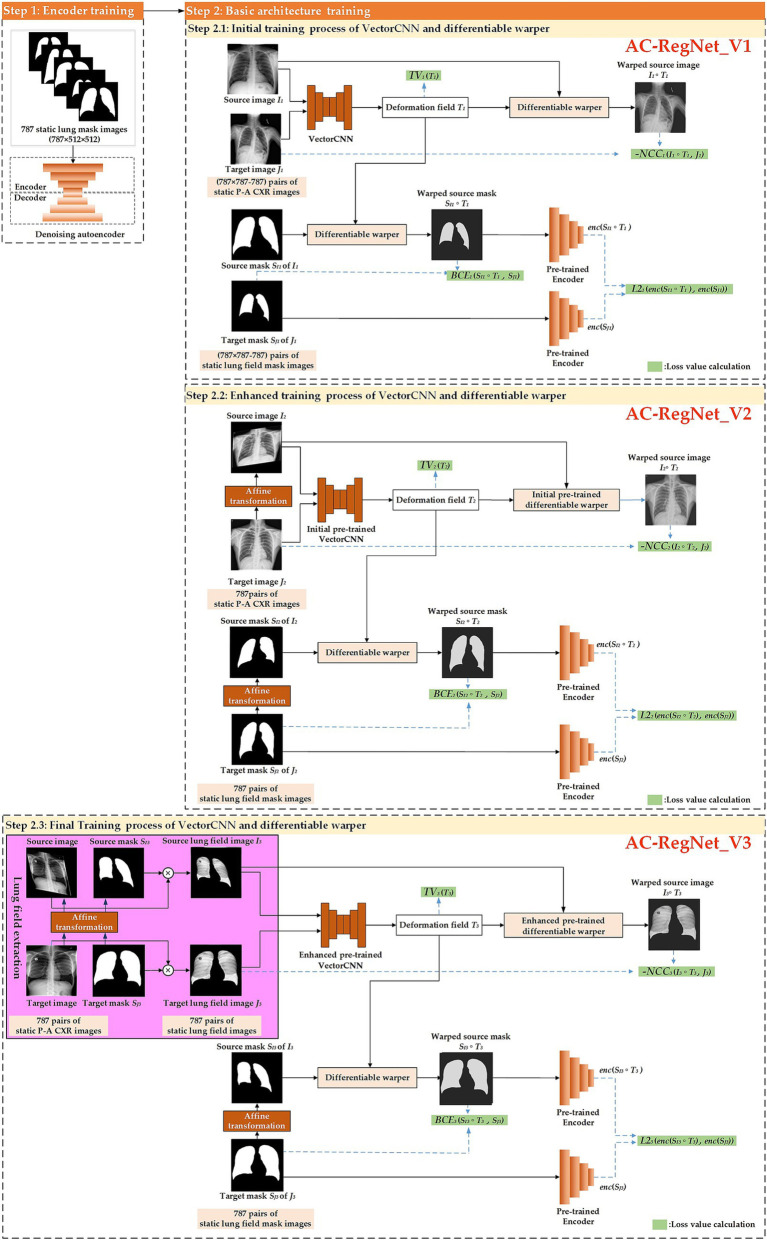
The meticulous training process of the registration network, designed to instill confidence in the method’s reliability.

Figure 3 shows the training process of the registration network (AC-RegNet), including the encoder and basic architecture training. Specifically, we follow the previous training method of AC-RegNet. First, 787 static CXR images and their lung field label images were used to train the anatomically constrained network [a kind of denoising autoencoders (Vincent et al., 2010)] on the lung field. Then, the encoder module of this pre-train anatomically constrained network participates in the training of VectorCNN and differentiable warper in the basic architecture of AC-RegNet. Since the registration task is to achieve dynamic lung field registration, the heart labels are discarded during the training of this anatomically constrained network.

Specifically, we propose a three-step registration training method to train the VectorCNN and differentiable warper of AC-RegNet. The three cascaded steps separately complete the initial, enhanced, and final training of the basic architecture of AC-RegNet based on 787 static P-A CXR images. First, any two pairs of these 787 static P-A CXR images (787 × 787–787 = 618,582) are combined. Then, they are configured as the source and target images for initially training the VectorCNN and differentiable warper (Step 2.1: Initial training process of VectorCNN and differentiable warper). Second, 787 static affine P-A CXR images are generated by affine transformation [a kind of data augmentation technique (Yang et al., 2024; Chlap et al., 2021)] of static P-A CXR images, respectively. Subsequently, 787 pairs of the source and target images are configured based on these 787 static P-A CXR images, and their affine images are used for enhanced training of the initial pre-trained VectorCNN and differentiable warper (Step 2.2: Enhanced training process of VectorCNN and differentiable warper). Third, 787 pairs of static lung field images are abstracted from their static P-A CXR images using the dynamic lung field extraction method in Section 2.2.2. Then, 787 static affine lung field images are generated by the affine transformation of 787 pairs of static lung field images. Similarly, these static lung field images and their affine images are used for the final training of the enhanced pre-trained VectorCNN and differentiable warper (Step 2.3: Final training process of VectorCNN and differentiable warper). Notably, AC-RegNet introduces an anatomically constrained network to produce anatomically plausible results during training. Specifically, the 618,582 (787 × 787–787) pairs of static lung field mask images participate in the initial training (Step 2.1 AC-RegNet_V1). Similarly, 787 pairs of lung field mask images and their affine images participate in the enhanced and final training (Step 2.2 AC-RegNet_V2 and Step 2.3 AC-RegNet_V3), respectively. It should be noted that the randomized parameters of affine transformation to each affine P-A CXR/lung field image and its corresponding affine lung field mask image should be consistent.

#### 2.2.4 Loss function

During each training step, the loss values are meticulously calculated to adjust the VectorCNN and differentiable warper parameters based on the following comprehensive loss function $Loss_{t}{\left(I_{t},J_{t},S_{\mathrm{It}},S_{Jt},T_{t}\right)}_{\mathrm{loss}}$ shown in Equation 1 (Yang et al., 2024; Mansilla et al., 2020; Balakrishnan et al., 2018; Ferrante et al., 2018; Yeap et al., 2023).


(1) $$\begin{array}{l} Loss_{t}{(}_{I_{t},J_{t},S_{\mathrm{It}},S_{J}t_{,}T_{t})\mathrm{loss}}\\ =-\lambda_{NCC}•NCC_{t}{(}_{I_{t}\circ T_{t},J_{t})\mathrm{loss}}+\lambda_{TV}•TV_{t}{(}_{T_{t})\mathrm{loss}}\\ +\lambda_{BCE}•BCE_{t}{(}_{S_{\mathrm{It}}\circ T_{t},S_{Jt})\mathrm{loss}}\\ +\lambda_{L2}•L2_{t}{(}_{enc(S_{\mathrm{It}}\circ T_{t}),enc(S_{Jt}))\mathrm{loss}} \end{array}$$

Where train step *t* = 1,2,3 represents the initial, enhanced, and final training process of VectorCNN and differentiable warper, respectively; parameters *I_t_*, *J_t_*, *S_It_*, *S_Jt_*, and *T_t_* separately represent the source image, target image, source mask, target mask, and deformation field at the *t*th train step; functions *NCC_t_* (), *TV_t_ ()*, *BCE_t_ ()*, and *L2_t_ ()* separately represent the Negative normalized cross-correlation (NCC) loss (Balakrishnan et al., 2018), total variation (TV) loss (Ferrante et al., 2018), binary cross-entropy (BCE) Loss (Yang et al., 2024; Mansilla et al., 2020), and L2 loss (Mansilla et al., 2020) at the *t*th train step. Meanwhile, the weight factors of functions *NCC_t_ ()*, *TV_t_ ()*, *BCE_t_ ()*, and *L2_t_ ()* are set to the previous default values. Therefore, the weight factors are: $\lambda_{NCC}$ =1.0, $\lambda_{TV}$ =5.0 × 10^−5^, $\lambda_{BCE}$ =1.0, $\lambda_{L2}$ =1.0 × 10^−1^. Last, *enc* represents the pre-trained encoder.

Specifically, Equation 2 presents the precise mathematical expression of NCC loss, which accurately describes the degree of correlation between the source images and their target images (Balakrishnan et al., 2018).


(2) $$-NCC_{t}{\left(I_{t}\circ T_{t},J_{t}\right)}_{\mathrm{loss}}=-\frac{cov\left(I_{t}\circ T_{t},J_{t}\right)}{\sqrt{Var\left(I_{t}\circ T_{t}\right)•Var\left(J_{t}\right)}+\varepsilon }$$

Where a non-zero factor *ε* is added to ensure that the denominator of NCC loss is not zero (*ε* = 1 × 10^−5)^, and functions *Cov* () and *Var* () separately represent the covariance and variance calculation.


(3) $$cov\left(I_{t}\circ T_{t},J_{t}\right)=E\left[\left(I_{t}\circ T_{t}-E\left(I_{t}\circ T_{t}\right)\right)\left(J_{t}-E\left(J_{t}\right)\right)\right]$$

(4) $$Var\left(I_{t}\circ T_{t}\right)=E\left[{\left(I_{t}\circ T_{t}-E\left(I_{t}\circ T_{t}\right)\right)}^{2}\right]$$

(5) $$Var\left(J_{t}\right)=E\left[{\left(J_{t}-E\left(J_{t}\right)\right)}^{2}\right]$$

Where function *E* () represents the expected value calculation.

Substitute these Equations 3–5 into Equation 2, obtaining Equation 6.


(6) $$-NCC_{t}{\left(I_{t}\circ T_{t},J_{t}\right)}_{\mathrm{loss}}=-\frac{E\left[\begin{array}{l} \left(I_{t}\circ T_{t}-E\left(I_{t}\circ T_{t}\right)\right)\\ \left(J_{t}-E\left(J_{t}\right)\right) \end{array}\right]}{\sqrt{\begin{array}{l} E\left[{\left(I_{t}\circ T_{t}-E\left(I_{t}\circ T_{t}\right)\right)}^{2}\right]•\\ E\left[{\left(J_{t}-E\left(J_{t}\right)\right)}^{2}\right] \end{array}}+\varepsilon }$$

The final simplified Equation 7 can be obtained using the expected linear property.


(7) $$-NCC_{t}{\left(I_{t}\circ T_{t},J_{t}\right)}_{\mathrm{loss}}=-\frac{E\left[\begin{array}{l} \left(I_{t}\circ T_{t}-E\left(I_{t}\circ T_{t}\right)\right)\\ \left(J_{t}-E\left(J_{t}\right)\right) \end{array}\right]}{\sqrt{\begin{array}{l} {}[E(\left(I_{t}\circ {T_{t}}^{2}\right)-{\left(E\left(I_{t}\circ T_{t}\right)\right)}^{2}]•\\ \left[E\left({J_{t}}^{2}\right)-{\left(E\left(J_{t}\right)\right)}^{2}\right] \end{array}}+\varepsilon }$$

The simplification process from Equations 6, 7 is shown in Equations 8–10.


(8) $$-NCC_{t}{\left(I_{t}\circ T_{t},J_{t}\right)}_{\mathrm{loss}}=-\frac{\begin{array}{l} E[(I_{t}\circ T_{t}-E\left(I_{t}\circ T_{t}\right)\\ \left(J_{t}-E\left(J_{t}\right)\right)] \end{array}}{\sqrt{\begin{array}{l} {E[((}^{I_{t}\circ T_{t})2}-2\left(I_{t}\circ T_{t}\right)•\\ E\left(I_{t}\circ T_{t}\right)+{(}^{E(I_{t}\circ T_{t}))2}]•\\ E[({J_{t}}^{2}-2J_{t}•E\left(J_{t}\right)+\\ {(}^{E(J_{t}))2}] \end{array}}+\varepsilon }$$

(9) $$-NCC_{t}{\left(I_{t}\circ T_{t},J_{t}\right)}_{\mathrm{loss}}=-\frac{\begin{array}{l} E[\left(I_{t}\circ T_{t}-E\left(I_{t}\circ T_{t}\right)\right)\\ \left(J_{t}-E\left(J_{t}\right)\right)] \end{array}}{\sqrt{\begin{array}{l} {}[E(\left({I_{t}\circ T_{t})}^{2}\right)-2E(\left(I_{t}\circ T_{t}\right)•\\ E\left(I_{t}\circ T_{t}\right))+E({E(I_{t}\circ T_{t}))}^{2}]•\\ {}[E\left({J_{t}}^{2}\right)-2E\left(J_{t}•E\left(J_{t}\right)\right)+\\ E({E(J_{t}))}^{2}] \end{array}}+\varepsilon }$$

(10) $$-NCC_{t}{\left(I_{t}\circ T_{t},J_{t}\right)}_{\mathrm{loss}}=-\frac{\begin{array}{l} E[\left(I_{t}\circ T_{t}-E\left(I_{t}\circ T_{t}\right)\right)\\ \left(J_{t}-E\left(J_{t}\right)\right)] \end{array}}{\sqrt{\begin{array}{l} {}[E({\left(I_{t}\circ T_{t})\right)}^{2}-2{(}^{E(I_{t}\circ T_{t}))2}+\\ {(}^{E(I_{t}\circ T_{t}))2}]•[E\left({J_{t}}^{2}\right)-2\\ {(}^{E(J_{t}))2}+{(}^{E(J_{t}))2}] \end{array}}+\varepsilon }$$

Specifically, Equation 11 shows the mathematical expression of TV loss, which smooths the source images’ registration images (Ferrante et al., 2018).


(11) $$\begin{array}{l} TV({T_{t})}_{\mathrm{loss}}=\left[\begin{array}{l} \mathrm{mean}(TV\left({T_{\mathrm{tx}})}_{\mathrm{loss}}\right)+\\ \mathrm{mean}(TV\left({T_{\mathrm{ty}})}_{\mathrm{loss}}\right) \end{array}\right]/2\\ =\left[\begin{array}{l} \mathrm{mean}\left(\sum_{i,j}^{n_{1}}\left|t_{i+1,j}-t_{i,j}\right|\right)+\\ \mathrm{mean}\left(\sum_{i,j}^{n_{2}}\left|t_{i,j+1}-t_{i,j}\right|\right) \end{array}\right]/2\\ =\left[\begin{array}{l} \left(\sum_{i,j}^{n_{1}}\left|t_{i+1,j}-t_{i,j}\right|\right)/n_{1}+\\ \left(\sum_{i,j}^{n_{2}}\left|t_{i,j+1}-t_{i,j}\right|\right)/n_{2} \end{array}\right]/2 \end{array}$$

Where function *mean ()* represents the average operation and this function |•| represents the absolute value operation; deformation field $T_{t}=\left(\begin{array}{ccc} t_{11} & \ldots & t_{1n_{1}}\\ \vdots & \ddots & \vdots \\ t_{n_{2}1} & \cdots & t_{n_{2}n_{1}} \end{array}\right)$ , $t_{i,j}\subset T_{t}$ ; n1 and n2 are the dimensions of deformation field $T_{t}$ at the *t*th train step; $T_{tx}$ and $T_{ty}$ separately represent the element of the x-axis and y-axis of the dimension of deformation field $T_{t}$ at the *t*th train step.

Specifically, Equation 12 shows the mathematical expression of BCE loss for calculating the difference between these target mask images and the warped mask images generated by the source mask images and differentiable warper (Yang et al., 2024; Mansilla et al., 2020).


(12) $$BCE_{t}{\left(S_{\mathrm{It}}\circ T_{t},S_{Jt}\right)}_{\mathrm{loss}}=\frac{1}{N}\underset{i}{\sum }-\left[\begin{array}{l} S_{Jti}•\log\left(S_{Iti}\circ T_{ti}\right)+\\ \left(1-S_{Jti}\right)•\\ \log\left(1-S_{Iti}\circ T_{ti}\right) \end{array}\right]$$

Where $S_{Jti}$ , $S_{Jti}$ , $T_{ti}$ , and *N* separately represent the *i*th source mask, target mask, deformation field at the *t*th train step, and the number of *i*.

Specifically, Equation 13 shows the mathematical expression of L2 loss for calculating the difference between these encoding target mask images and their encoding warped mask images (Mansilla et al., 2020).


(13) $$\begin{array}{l} L2_{t}{\left(enc\left(S_{\mathrm{It}}\circ T_{t}\right),enc\left(S_{Jt}\right)\right)}_{\mathrm{loss}}=\mathrm{mean}\left(\|\begin{array}{l} enc\left(S_{\mathrm{It}}\circ T_{t}\right)-\\ enc\left(S_{Jt}\right) \end{array}{\|}_{2}^{2}\right)\\ =\overset{n}{\underset{j}{\sum }}\left(\left(\overset{n}{\underset{i,j=1}{\sum }}{\left(st_{i,j}-s_{i,j}\right)}^{2}\right)/n\right) \end{array}$$

Where function *mean ()* represents the average operation and this function ${\left\|\right\|}_{2}$ represents the 2-norm operation; the encoding warped mask images $enc\left(S_{\mathrm{It}}\circ T_{t}\right)=\left(\begin{array}{ccc} st_{11} & \ldots & st_{1n}\\ \vdots & \ddots & \vdots \\ st_{n1} & \cdots & st_{nn} \end{array}\right)$ , $st_{i,j}\subset enc\left(S_{\mathrm{It}}\circ T_{t}\right)$ ; the encoding target mask images $enc\left(S_{Jt}\right)=\left(\begin{array}{ccc} s_{11} & \ldots & s_{1n}\\ \vdots & \ddots & \vdots \\ s_{n1} & \cdots & s_{nn} \end{array}\right)$ , $s_{i,j}\subset enc\left(S_{Jt}\right)$ ; *n* × *n* is the size of the encoding warped mask images or target mask images.

Finally, this final-train network is applied to register the dynamic lung field images of 512 × 512 P-A CXR images in the dataset D6.

## 3 Experiments

This section conducts comprehensive ablation and comparison studies based on the above materials and methods. The purpose of these studies is to reflect the scientific validity and superior performance of our proposed registration model (AC-RegNet_V3) in a variety of scenarios.

### 3.1 Experimental design

**Figure 4:**
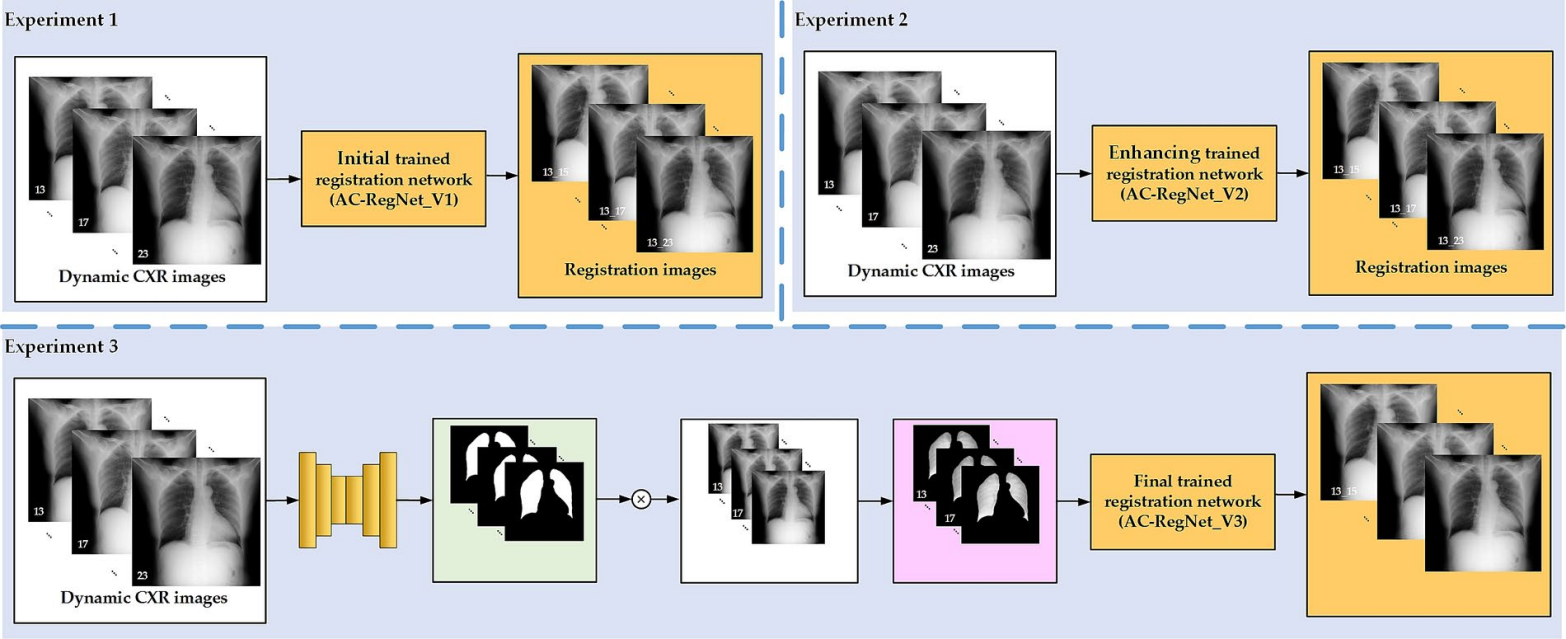
Experimental design for the ablation study.

Figure 4 shows the experimental design for the ablation study to compare the effects of different steps (AC-RegNet_V1, AC-RegNet_V2, AC-RegNet_V3). Specifically, the fixed lung filed mask images are generated from the dynamic P-A CXR images based on a pre-trained lung field segmentation network [U-Net (Ronneberger et al., 2015)]. Besides, experiments 1 and 2 register any two pairs of these 11 dynamic P-A CXR images. Unlike experiments 1 and 2, experiment 3 registers any two pairs of these dynamic lung field images abstracted from the dynamic P-A CXR images based on their lung field mask images.

**Figure 5:**
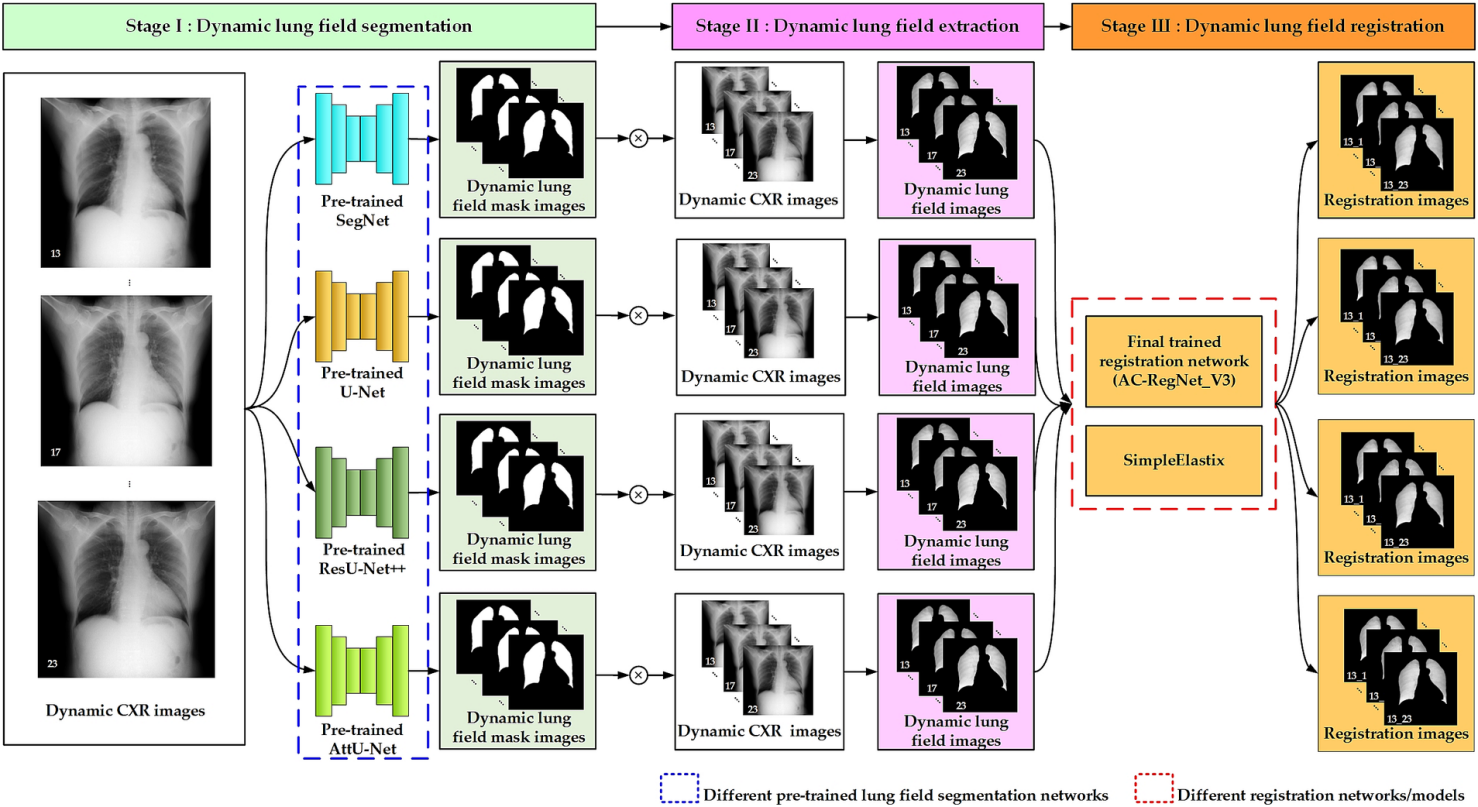
Experimental design for the comparing study.

In addition, Figure 5 shows the experimental design for the comparing study to compare the performance of our proposed registration model and the SimpleElastix. Previous research confirmed that the evaluation metrics of the AC-RegNet have surpassed the AE-RegNet, CE-RegNet, and RegNet (Mansilla et al., 2020). Therefore, the evaluation metrics among these registration models above will not be compared again in the experimental section. Specifically, four pre-trained lung field segmentation networks, SegNet (Badrinarayanan et al., 2017), U-Net (Ronneberger et al., 2015)], ResU-Net++ (Yang et al., 2021; Jha et al., 2019), and AttU-Net (Wang et al., 2021), to compare the effects of different lung field segmentation networks on the proposedAC-RegNet_V3. Besides, the widely used unsupervised technology for medical image registration, SimpleElastix (Marstal et al., 2016) with the affine transform, is also compared with the proposed AC-RegNet_V3. Specifically, these four pre-trained lung field segmentation networks are separately applied to segment the lung fields of 11 sets of dynamic P-A CXR images, obtaining four sets of dynamic lung field mask images in Stage І. Then, four groups of dynamic lung field images are abstracted from the dynamic P-A CXR images based on their lung field mask images in stage ІІ. Ultimately, 110 pairs of the source and target images are generated by combining any two pairs of these 11 sets of dynamic lung field images in each group. Last, the AC-RegNet_V3 and SimpleElastix are applied separately to register the dynamic lung field image groups in stage Ш.

### 3.2 Evaluation metrics

Our previous study has displayed the evaluation metrics of these four pre-trained segmentation networks (Yang et al., 2024; Yang et al., 2024). Therefore, this section mainly presents and compares the registration evaluation metrics of these dynamic CXRs, lung fields, and registration images.

After completing the registration of 110 pairs of the source and target images, four stand evaluation metrics, dice similarity coefficient (DSC) (Mansilla et al., 2020; Yeap et al., 2023), Hausdorff distance (HD) (Yang et al., 2024), average symmetric surface distance (ASSD) (Mansilla et al., 2020) and mean squared distance (MSD) (Rahunathan et al., 2005), are used to evaluate these dynamic CXR, lung field, and registration images. Besides, unlike medical images of non-deformable tissues such as brain and bone images (Guo et al., 2022; Guo et al., 2022; Guo et al., 2022; Yang and Guo, 2024; Chang and Lee, 2021; Guo et al., 2022), the lung fields in dynamic CXR images undergo anatomical folding during respiration. Therefore, the Jacobian determinant (Kuang, 2019) is excluded from this study to evaluate the registration performance.

The DSC, HD, and ASSD focus on changes in lung field morphology based on their mask, while the evaluation metric MSD focuses on changes in the CXR or lung field image. Equations 14–17 show the mathematical expression of these four stand evaluation metrics (Yang et al., 2024; Mansilla et al., 2020; Yeap et al., 2023; Rahunathan et al., 2005).


(14) $$DSC=2\times \frac{\left|I_{\mathrm{fixed}}\cap I_{\mathrm{registered}/\mathrm{moving}}\right|}{\left|I_{\mathrm{fixed}}|+|I_{\mathrm{registered}/\mathrm{moving}}\right|}$$

(15) $$\begin{array}{l} HD=\max\left(d_{I_{\mathrm{fixed}}I_{\mathrm{registered}/\mathrm{moving}}},d_{I_{\mathrm{registered}/\mathrm{moving}}I_{\mathrm{fixed}}}\right)\\ =\max\left(\left\{\begin{array}{l} \max_{x\in I_{\mathrm{fixed}}}\min_{y\in I_{\mathrm{registered}/\mathrm{moving}}}\|x,y\|,\\ \max_{y\in I_{\mathrm{registered}/\mathrm{moving}}}\min_{x\in I_{\mathrm{fixed}}}\|x,y\| \end{array}\right\}\right) \end{array}$$

(16) $$\begin{array}{l} \mathrm{ASSD}=\frac{1}{|I_{\mathrm{fixed}}|+I_{\mathrm{registered}/\mathrm{moving}}}\times \\ \left(\begin{array}{l} \underset{\left(x,y\right)\in I_{\mathrm{fixed}}}{\sum }\|\left(x,y\right),I_{\mathrm{fixed}}\|+\\ \underset{\left(x,y\right)\in I_{\mathrm{registered}/\mathrm{moving}}}{\sum }\|\left(x,y\right),I_{\mathrm{registered}/\mathrm{moving}}\| \end{array}\right) \end{array}$$

(17) $$MSD=\frac{1}{N}\underset{\left(x,y\right)}{\sum }{\left[I_{\mathrm{fixed}}\left(x,y\right)-I_{\mathrm{registered}/\mathrm{moving}}\left(x,y\right)\right]}^{2}$$

Where $I_{\mathrm{fixed}}$ represents the fixed lung filed mask image of the $I_{\mathrm{fixed}}\left(x,y\right)$ , and $I_{\mathrm{fixed}}\left(x,y\right)$ represents the fixed CXR image. Besides, $I_{\mathrm{registered}/\mathrm{moving}}$ represents the registered/moving lung filed mask image of $I_{\mathrm{registered}/\mathrm{moving}}\left(x,y\right)$ , and $I_{\mathrm{registered}/\mathrm{moving}}\left(x,y\right)$ represents the registered/moving CXR image. Last, this function ‖‖ represents the 2-norm operation, and *N* represents the pixel number of the fixed or registered/moving CXR image.

## 4 Results

This section comprehensively compares and presents the registration results of dynamic lung field images based on the above materials and methods.

### 4.1 Ablation study results

**Figure 6:**
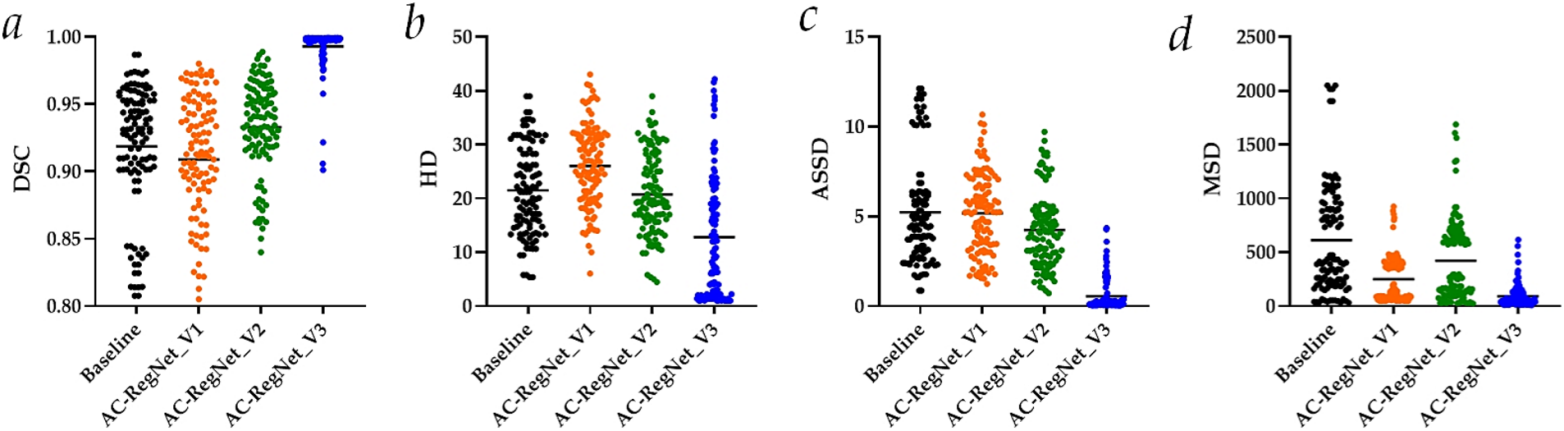
Visual distribution of the evaluation metrics of the AC-RegNet_V1-V3 in the ablation study. **(a)** Dice similarity coefficient (DSC); **(b)** Hausdorff distance (HD); **(c)** Average symmetric surface distance (ASSD); **(d)** Mean squared distance (MSD).

Table 1 separately shows the descriptive statistics on the evaluation metrics of the 110 pairs of images based on the ablation study. Besides, Figure 6 shows the visual distribution of the evaluation metrics of the Baseline and AC-RegNet_V1-V3 in the ablation study, respectively.

**Table 1:** The evaluation metrics of the ablation study.

Evaluation metrics	Network			110 pairs of images
	Segmentation	Lung field extraction	Registration	
DSC (mean ± SD*)	×	×	×	0.919 (0.808–0.987) ± 0.047
	×	×	AC-RegNet_V1	0.909 (0.762–0.980) ± 0.049
	×	×	AC-RegNet_V2	0.933 (0.840–0.989) ± 0.034
	U-Net [26]	✓	AC-RegNet_V3	0.993 (0.901–1.000) ± 0.016
HD (mean ± SD*)	×	×	×	21.506 (5.385–39.000) ± 8.243
	×	×	AC-RegNet_V1	26.074 (6.083–43.046) ± 7.577
	×	×	AC-RegNet_V2	20.755 (4.472–39.000) ± 7.642
	U-Net [26]	✓	AC-RegNet_V3	12.813 (1.000–42.202) ± 11.460
ASSD (mean ± SD*)	×	×	×	5.230 (0.866–12.130) ± 2.947
	×	×	AC-RegNet_V1	5.197 (1.245–10.690) ± 2.253
	×	×	AC-RegNet_V2	4.243 (0.719–9.270) ± 2.116
	U-Net [26]	✓	AC-RegNet_V3	0.550 (0.035–4.361) ± 0.911
MSD (mean ± SD*)	×	×	×	612.654 (32.924–2050.390) ± 503.383
	×	×	AC-RegNet_V1	250.506 (46.376–926.845) ± 212.108
	×	×	AC-RegNet_V2	422.766 (23.824–1688.810) ± 381.697
	U-Net [26]	✓	AC-RegNet_V3	92.878 (9.438–616.990) ± 106.752

* SD, standard deviation.

Specifically, DSC (mean ± SD), HD (mean ± SD), ASSD (mean ± SD), and MSD (mean ± SD) of these 110 pairs of dynamic CXR images (Baseline in Figure 6) are 0.919 ± 0.047, 21.506 ± 8.243, 5.230 ± 2.947, and 612.654 ± 503.383, respectively. Meanwhile, the evaluation metrics of the 110 pairs of dynamic CXR images based on AC-RegNet_V1 achieve the DSC (mean ± SD) of 0.909 ± 0.049, HD (mean ± SD) of 26.074 ± 7.577, and ASSD (mean ± SD) of 5.197 ± 2.253, and MSD (mean ± SD) of 250.506 ± 212.108. The evaluation metrics of the 110 pairs of dynamic CXR images based on AC-RegNet_V2 achieve the DSC (mean ± SD) of 0.933 ± 0.034, HD (mean ± SD) of 20.755 ± 7.642, and ASSD (mean ± SD) of 4.243 ± 2.116, and MSD (mean ± SD) of 422.766 ± 381.697. The evaluation metrics of the 110 pairs of dynamic CXR images based on AC-RegNet_V3 achieve the DSC (mean ± SD) of 0.993 ± 0.016, HD (mean ± SD) of 12.813 ± 11.460, and ASSD (mean ± SD) of 0.550 ± 0.911, and MSD (mean ± SD) of 92.878 ± 106.752.

Compared with the evaluation metrics of the Baseline, only all the evaluation metrics of the AC-RegNet_V_3 have significantly improved (*p*-value < 0.0001). Specifically, compared with the mean DSC of the Baseline, this evaluation metric of the AC-RegNet_V1, AC-RegNet_V2, and AC-RegNet_V3 is improved by −0.010 (↓), 0.014 (↑), and 0.074 (↑), respectively. Besides, compared with the mean HD of the Baseline, this evaluation metric of the AC-RegNet_V1, AC-RegNet_V2, and AC-RegNet_V3 is improved by 4.568 (↓), −0.751 (↑), and − 8.693 (↑), respectively. Meanwhile, compared with the mean ASSD of the Baseline, this evaluation metric of the AC-RegNet_V1, AC-RegNet_V2, and AC-RegNet_V3 is improved by −0.033 (↑), −0.987 (↑), and − 4.680 (↑), respectively. Last, compared with the mean MSD of the Baseline, this evaluation metric of the AC-RegNet_V1, AC-RegNet_V2, and AC-RegNet_V3 is improved by −362.148 (↑), −189.888 (↑), and − 519.776 (↑), respectively.

Compared with the evaluation metrics of the AC-RegNet_V1, all these evaluation metrics of the AC-RegNet_V2 and AC-RegNet_V_3 have significantly improved (*p*-value < 0.0001). Specifically, compared with the mean DSC of the AC-RegNet_V1, this evaluation metric of the AC-RegNet_V2 and AC-RegNet_V3 is improved by 0.024 (↑) and 0.084 (↑), respectively. Besides, compared with the mean HD of the AC-RegNet_V1, this evaluation metric of the AC-RegNet_V2 and AC-RegNet_V3 is improved by −5.319 (↑) and − 13.261 (↑), respectively. Meanwhile, compared with the mean ASSD of the AC-RegNet_V1, this evaluation metric of the AC-RegNet_V2 and AC-RegNet_V3 is improved by −0.954 (↑) and − 4.647 (↑), respectively. Last, compared with the mean MSD of the AC-RegNet_V1, this evaluation metric of the AC-RegNet_V2 and AC-RegNet_V3 is improved by 172.26 (↓) and − 157.628 (↑), respectively.

Compared with the evaluation metrics of the AC-RegNet_V2, all these evaluation metrics of the AC-RegNet_V3 have significantly improved (*p*-value < 0.0001). Specifically, compared with the mean DSC of the AC-RegNet_V2, this evaluation metric of the AC-RegNet_V3 is improved by 0.06 (↑). Besides, compared with the mean HD of the AC-RegNet_V2, this evaluation metric of the AC-RegNet_V3 is improved by −7.942 (↑). Meanwhile, compared with the mean ASSD of the AC-RegNet_V2, this evaluation metric of the AC-RegNet_V3 is improved by −3.693 (↑). Last, compared with the mean MSD of the AC-RegNet_V2, this evaluation metric of the AC-RegNet_V3 is improved by −329.888 (↓).

### 4.2 Comparing study results

Table 2 shows the descriptive statistics on the evaluation metrics of the 110 pairs of dynamic lung field registration images based on the comparing study. Meanwhile, Figure 7 shows the visual distribution of the evaluation metrics of the SimpleElastix and AC-RegNet_V3 based on the four pre-trained segmentation networks. Last, Figures 8–11 show the visual differences among the dynamic CXR, lung field, and registration images of the source image (13) and target images (14–23) based on the SimpleElastixSegNet and AC-RegNet_V3 with the different pre-trained lung field segmentation networks.

**Table 2:** The evaluation metrics of the comparing study.

Evaluation metrics	Network			110 pairs of images
	Segmentation	Lung field extraction	Registration	
DSC (mean ± SD*)	SegNet [25]	✓	SimpleElastix [10]	0.960 (0.926–0.983) ± 0.014
	U-Net [26]	✓	SimpleElastix [10]	0.959 (0.926–0.983) ± 0.020
	ResU-Net ++ [17]	✓	SimpleElastix [10]	0.963 (0.918–0.989) ± 0.019
	AttU-Net [18]	✓	SimpleElastix [10]	0.962 (0.924–0.987) ± 0.017
	SegNet [25]	✓	AC-RegNet_V3	0.991 (0.894–0.999) ± 0.018
	U-Net [26]	✓	AC-RegNet_V3	0.993 (0.901–1.000) ± 0.016
	ResU-Net ++ [17]	✓	AC-RegNet_V3	0.993 (0.898–1.000) ± 0.017
	AttU-Net [18]	✓	AC-RegNet_V3	0.993 (0.905–1.000) ± 0.016
HD (mean ± SD*)	SegNet [25]	✓	SimpleElastix [10]	17.874 (7.071–37.947) ± 8.186
	U-Net [26]	✓	SimpleElastix [10]	19.147 (4.472–39.217) ± 9.136
	ResU-Net ++ [17]	✓	SimpleElastix [10]	17.478 (4.000–36.674) ± 8.818
	AttU-Net [18]	✓	SimpleElastix [10]	17.651 (6.403–37.577) ± 7.486
	SegNet [25]	✓	AC-RegNet_V3	12.512 (1.000–42.579) ± 11.338
	U-Net [26]	✓	AC-RegNet_V3	12.813 (1.000–42.202) ± 11.460
	ResU-Net ++ [17]	✓	AC-RegNet_V3	12.449 (1.000–43.139) ± 11.450
	AttU-Net [18]	✓	AC-RegNet_V3	13.661 (1.000–43.278) ± 11.760
ASSD (mean ± SD*)	SegNet [25]	✓	SimpleElastix [10]	2.590 (1.115–4.795) ± 0.928
	U-Net [26]	✓	SimpleElastix [10]	2.653 (0.762–5.812) ± 1.279
	ResU-Net ++ [17]	✓	SimpleElastix [10]	2.506 (0.757–5.416) ± 1.278
	AttU-Net [18]	✓	SimpleElastix [10]	2.525 (0.892–4.999) ± 1.126
	SegNet [25]	✓	AC-RegNet_V3	0.654 (0.065–4.665) ± 1.025
	U-Net [26]	✓	AC-RegNet_V3	0.550 (0.035–4.361) ± 0.911
	ResU-Net ++ [17]	✓	AC-RegNet_V3	0.572 (0.025–4.825) ± 0.992
	AttU-Net [18]	✓	AC-RegNet_V3	0.564 (0.019–4.702) ± 0.988
MSD (mean ± SD*)	SegNet [25]	✓	SimpleElastix [10]	559.098 (156.463–1212.521) ± 225.013
	U-Net [26]	✓	SimpleElastix [10]	577.797 (127.297–1200.305) ± 269.190
	ResU-Net ++ [17]	✓	SimpleElastix [10]	548.189 (116.112–1183.068) ± 254.528
	AttU-Net [18]	✓	SimpleElastix [10]	559.652 (131.187–1117.780) ± 247.021
	SegNet [25]	✓	AC-RegNet_V3	103.718 (13.923–658.279) ± 116.340
	U-Net [26]	✓	AC-RegNet_V3	92.878 (9.438–616.990) ± 106.752
	ResU-Net ++ [17]	✓	AC-RegNet_V3	94.750 (8.655–630.870) ± 94.750
	AttU-Net [18]	✓	AC-RegNet_V3	92.028 (8.731–607.461) ± 109.771

* SD, standard deviation.

**Figure 7:**
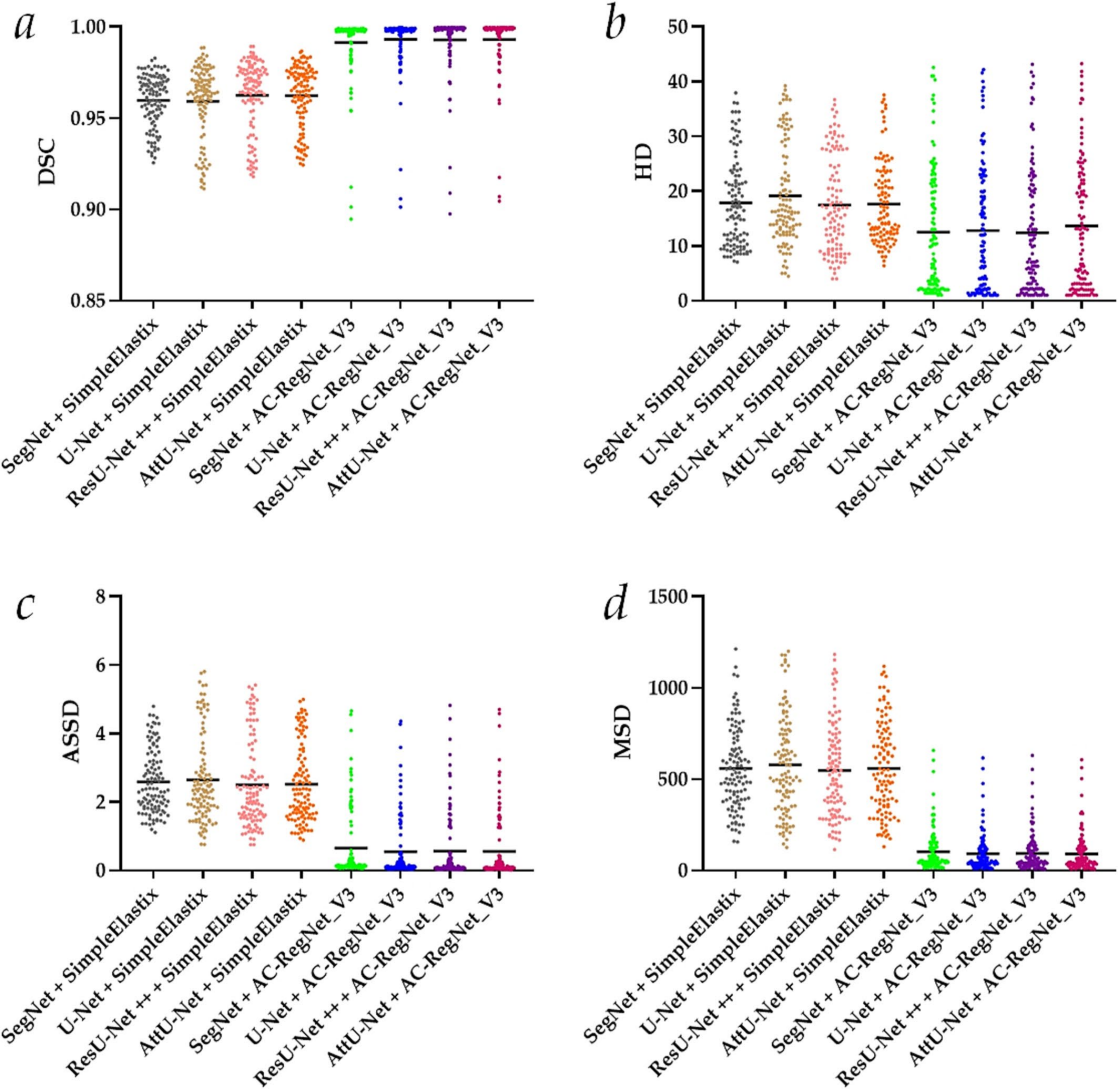
Visual distribution of the evaluation metrics of the SimpleElastix and AC-RegNet_V3 based on the four pre-trained segmentation networks. **(a)** Dice similarity coefficient (DCS); **(b)** Hausdorff distance (HD); **(c)** Average symmetric surface distance (ASSD); **(d)** Mean squared distance (MSD).

**Figure 8:**
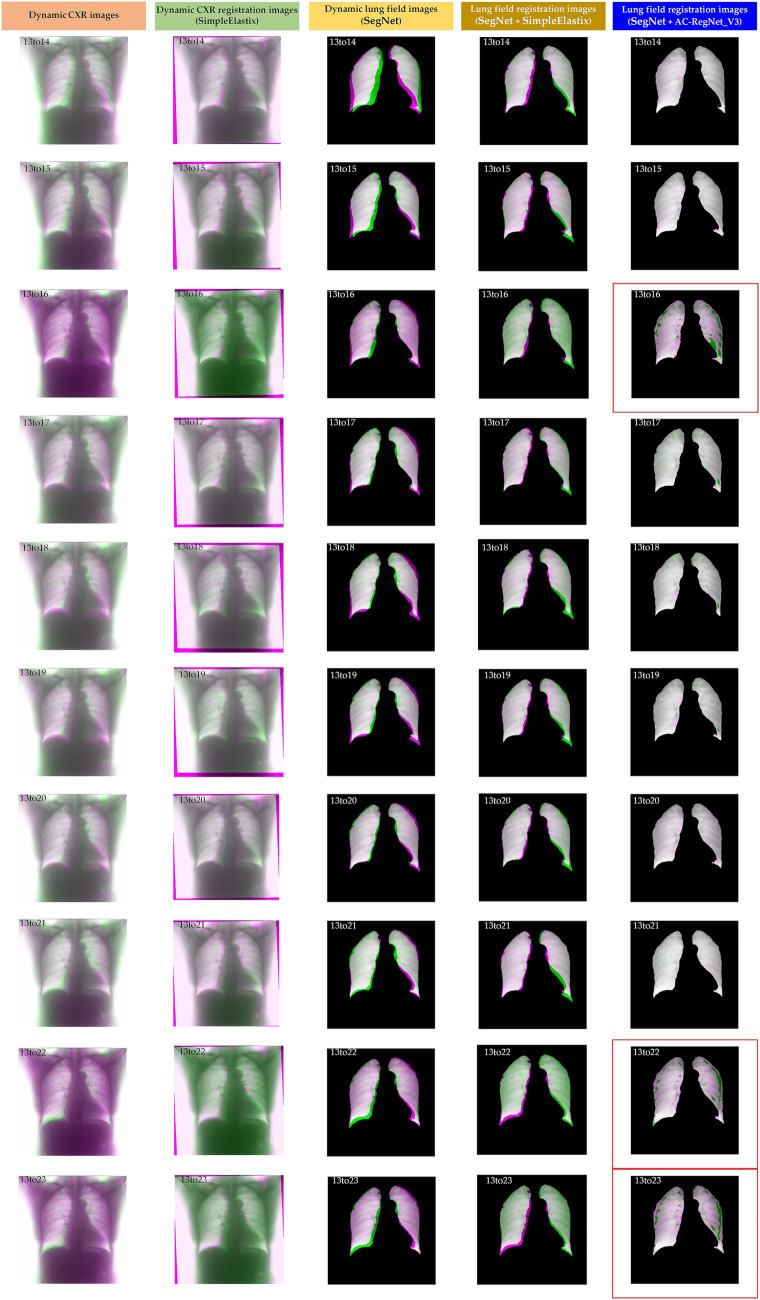
Visual differences among the dynamic CXR, lung field, and registration images of the source image (13) and target images (14–23) based on the SimpleElastixand AC-RegNet_V3 with the SegNet.

**Figure 9:**
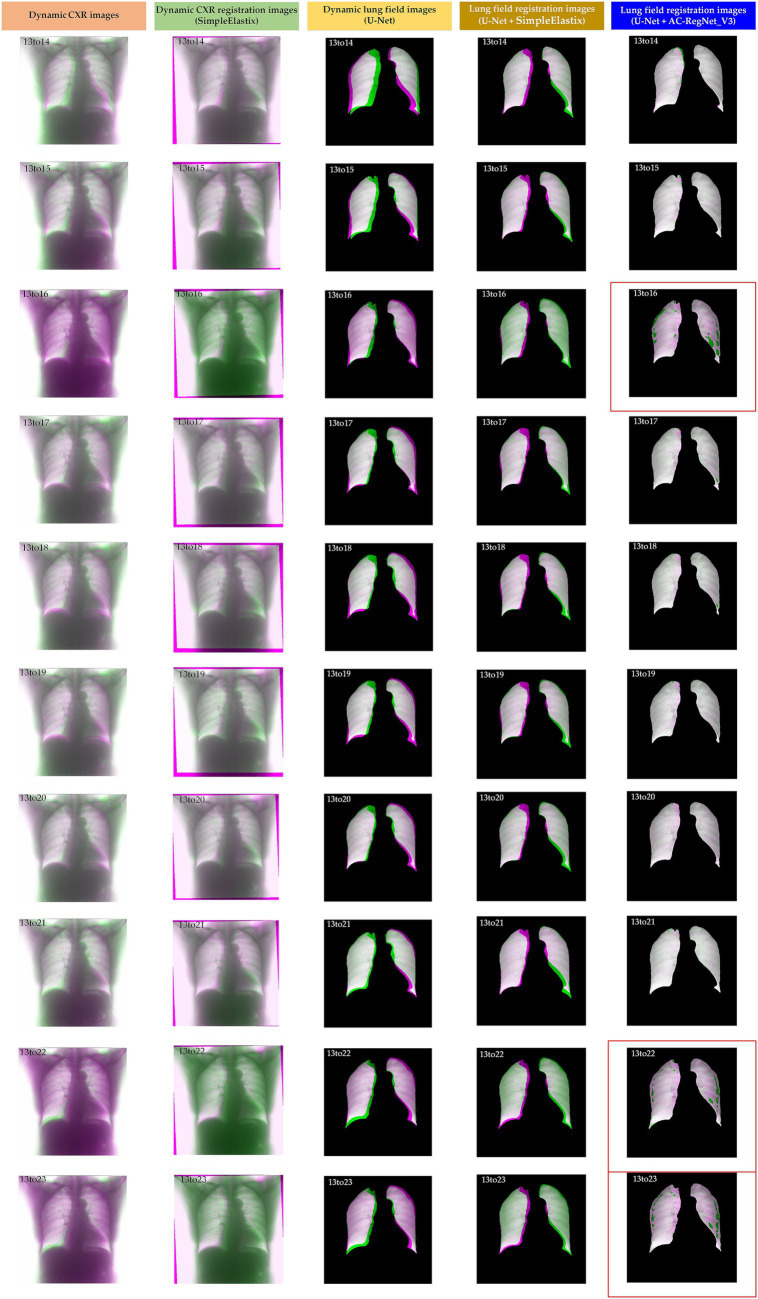
Visual differences among the dynamic CXR, lung field, and registration images of the source image (13) and target images (14–23) based on the SimpleElastix and AC-RegNet_V3 with the Unet.

**Figure 10:**
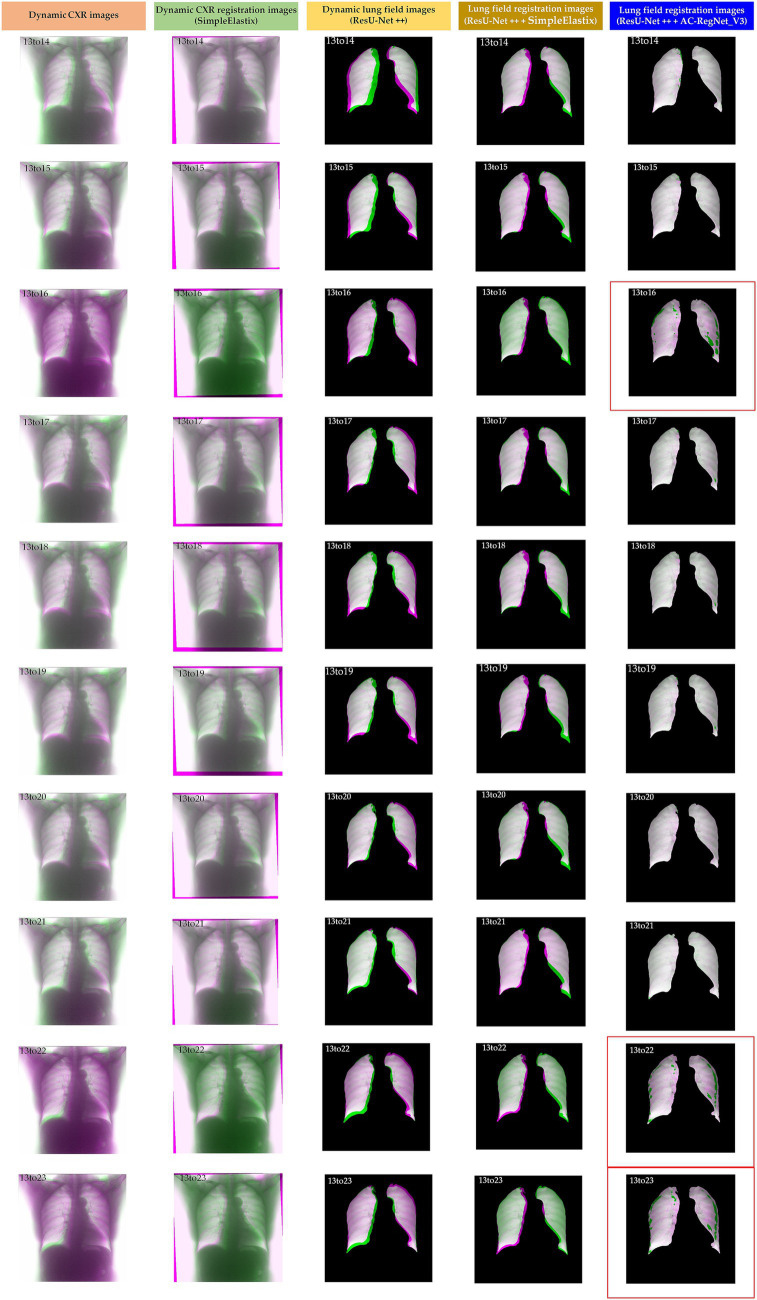
Visual differences among the dynamic CXR, lung field, and registration images of the source image (13) and target images (14–23) based on the SimpleElastix and AC-RegNet_V3 with the ResU-Net ++.

**Figure 11:**
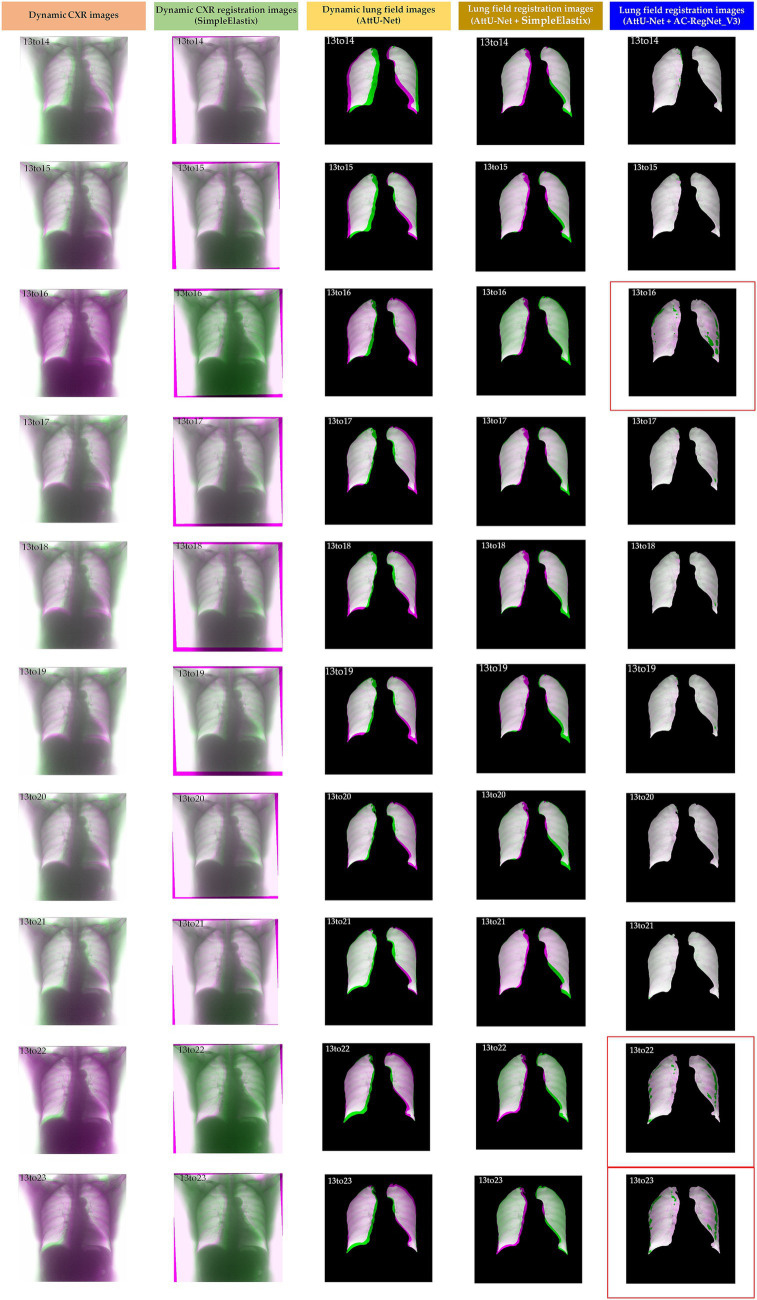
Visual differences among the dynamic CXR, lung field, and registration images of the source image (13) and target images (14–23) based on the SimpleElastix and AC-RegNet_V3 with the AttU-Net.

First, the DSC (mean ± SD) of these 110 pairs of dynamic lung field and registration images based on the SimpleElastixSegNet with the SegNet, U-Net, ResU-Net ++, and AttU-Net are 0.960 ± 0.014, 0.959 ± 0.020, 0.963 ± 0.019, and 0.962 ± 0.017, respectively. Meanwhile, the DSC (mean ± SD) of these 110 pairs of dynamic lung field and registration images based on the AC-RegNet_V3with the SegNet, U-Net, ResU-Net ++, and AttU-Net are 0.991 ± 0.018, 0.993 ± 0.016, 0.993 ± 0.017, and 0.993 ± 0.016, respectively. Compared with the mean DSC of 0.961 [(0.96 + 0.959 + 0.963 + 0.962)/4] based on the SimpleElastixSegNet, this mean evaluation metric of 0.993 based on the AC-RegNet_V3 is improved by 0.0315 (↑).

Second, the HD (mean ± SD) of these 110 pairs of dynamic lung field and registration images based on the SimpleElastixSegNet with the SegNet, U-Net, ResU-Net ++, and AttU-Net are 17.874 ± 8.186, 19.147 ± 9.136, 17.478 ± 8.818, and 17.651 ± 7.486, respectively. Meanwhile, the HD (mean ± SD) of these 110 pairs of dynamic lung field and registration images based on the AC-RegNet_V3with the SegNet, U-Net, ResU-Net ++, and AttU-Net are 12.512 ± 11.338, 12.813 ± 11.460, 12.449 ± 11.450, and 13.661 ± 11.760, respectively. Compared with the mean HD of 18.038 based on the SimpleElastixSegNet, this mean evaluation metric of 12.859 based on the AC-RegNet_V3 is improved by −5.179 (↑).

Third, the ASSD (mean ± SD) of these 110 pairs of dynamic lung field and registration images based on the SimpleElastixSegNet with the SegNet, U-Net, ResU-Net ++, and AttU-Net are 2.590 ± 0.928, 2.653 ± 1.279, 2.506 ± 1.278, and 2.525 ± 1.126, respectively. Meanwhile, the ASSD (mean ± SD) of these 110 pairs of dynamic lung field and registration images based on the AC-RegNet_V3with the SegNet, U-Net, ResU-Net ++, and AttU-Net are 0.654 ± 1.025, 0.550 ± 0.911, 0.572 ± 0.992, and 0.564 ± 0.988, respectively. Compared with the mean ASSD of 2.569 based on the SimpleElastixSegNet, this mean evaluation metric of 0.585 based on the AC-RegNet_V3 is improved by −1.984 (↑).

Last, the MSD (mean ± SD) of these 110 pairs of dynamic lung field and registration images based on the SimpleElastixSegNet with the SegNet, U-Net, ResU-Net ++, and AttU-Net are 559.098 ± 225.013, 577.797 ± 269.190, 548.189 ± 254.528, and 559.652 ± 247.021, respectively. Meanwhile, the MSD (mean ± SD) of these 110 pairs of dynamic lung field and registration images based on the AC-RegNet_V3with the SegNet, U-Net, ResU-Net ++, and AttU-Net are 103.718 ± 116.340, 92.878 ± 106.752, 94.750 ± 94.750, and 92.028 ± 109.771, respectively. Compared with the mean MSD of 561.184 based on the SimpleElastixSegNet, this mean evaluation metric of 95.844 based on the AC-RegNet_V3 is improved by −465.341 (↑).

Figures 8–11 show the visual differences among the dynamic CXR, lung field, and registration images of the source image (13) and target images (14–23) based on the SimpleElastix and AC-RegNet_V3 with the different pre-trained lung field segmentation networks. The registration effect of the proposed AC-RegNet_V3 comprehensively surpasses that of the SimpleElastix. Visually, the source image (13) can be well aligned with target images (14–23) based on each lung field segmentation network with AC-RegNet_V3. However, it is easy to observe a certain degree of tearing on the registration images (13_16, 13_22, and 13_23 marked by the red box) based on each lung field segmentation network with AC-RegNet_V3.

## 5 Discussion

This section conducts the following discussion and points out this study’s limitations based on the experimental results.

### 5.1 Dynamic lung field registration driven by automatic segmentation technology

The automatic organ segmentation task based on CNN is the foundation and key to achieving quantitative analysis of regions of interest (ROI) in medical images, such as 3D chest CT images, magnetic resonance angiography images, and cardiac MRI images, etc. (Yang et al., 2024; Yang et al., 2024; Deng et al., 2024; Wang et al., 2024; Yang et al., 2021; Zaman et al., 2024; Zeng et al., 2023; Duan et al., 2023). Similarly, this critical task also exists in the CXR images for ROI segmentation of the lung field.

Accurate lung field registration results may be more favored and exciting than the registration results of the entire CXR images for dynamic lung field analysis in clinical practice. The effective and accurate lung field segmentation of dynamic P-A CXR images will drive the development of dynamic lung field registration for clinical quantitative analysis. Meanwhile, the automatic lung field segmentation of the P-A CXR images is a data diversity problem, not a methodology problem (Yang et al., 2024). Therefore, our previous study trained several robust and standard segmentation networks of pathological lungs based on the CNN with cross-center static P-A CXR images and their diversity of disease. Besides, the data augmentation technology is applied to enrich the training set of the static P-A CXR images and relieve the engineering problem of generalization in these lung field segmentation networks (Yang et al., 2024; Chlap et al., 2021; Kiruthika and Khilar, 2024; Hasan and Abdulazeez, 2024). These robust and standard segmentation networks provide optional models for lung field segmentation in dynamic P-A chest CXR images. Based on the above, the proposal of these segmentation networks has laid a solid foundation for the lung field segmentation of cross-center dynamic P-A CXR images, allowing the subsequent registration of the dynamic lung fields. Actually, the registration evaluation metrics further indicate that these segmentation networks can meet the requirements for registering dynamic lung field images.

### 5.2 Dynamic lung field registration driven by static P-A CXR images

Compared with static P-A CXR images, dynamic CXR images have not been widely collected. Meanwhile, the lung undergoes a non-rigid and complex process of contraction and expansion during breathing (Santhanam, 2006). Compared to other static organs or tissues (such as the brain, bones, etc.), these non-rigid and complex deformations present significant challenges for lung field registration. Therefore, the limited number of dynamic CXR images and the non-rigid and complex deformations in the dynamic lung field make registering the dynamic lung fields challenging.

Based on the above, this paper fully utilizes static P-A CXR images to simulate the non-rigid and complex deformations in the dynamic lung field during the enhanced and final training of the basic architecture of AC-RegNet. Although AC-RegNet_V1 can produce certain deformations in dynamic images collected from the same person’s breathing process, it lacks learning from dynamic CXR images during training, resulting in its inability to register dynamic CXR images well. When the enhanced and final training of the basic architecture of AC-RegNet, we are inspired by these lung field segmentation networks above and subsequently apply the affine transformation technology (Yang et al., 2024; Chlap et al., 2021), originally used for data augmentation for training segmentation networks, to simulate lung respiratory changes in the static P-A CXR and lung field images. Compared with AC-RegNet_V1, even if simulated dynamic images are added during the training process of AC-RegNet_2, the network needs to consider the registration of the entire CXR image, which imposes a great registration burden on the network. However, AC-RegNet_V3 eliminates this registration burden by focusing only on the lung fields. The significant improvement in registration evaluation metrics also benefits from the simulated changes in lung respiration mentioned above. Meanwhile, when the lung field changes too much during respiration, the lung field in the registration images may tear due to significant deformation of the source image for alignment with the target image. In addition, there is tearing in the lung field in the registered images, leading to a reasonably great value in HD of the evaluation metrics. However, we believe that this tearing is inevitable.

### 5.3 Providing the possibility for quantitative analysis of dynamic lung fields

The proposed fully automatic registration pipeline effectively registers dynamic lung field images to maintain lung morphology alignment during respiration. Thus, this may become a valuable tool for further quantitative analysis of dynamic lung fields, such as pulmonary airflow detection (Jiang et al., 2024) and air trapping location (Zhang et al., 2024).

Specifically, the most direct way is to use registered images from adjacent time points to detect the trajectory of lung field movement, thereby revealing the trajectory rules in the respiratory process. Besides, like the air trapping location based on expiratory and inspiratory CT images (Deng et al., 2024; Wang et al., 2024), a pair of lung field images with the maximum and minimum areas can be determined during a respiratory cycle. Then, the registration images based on this pair of lung field images can be used to locate the air trapping, which may provide a new avenue for diagnosing chronic obstructive pulmonary disease based on dynamic CXR images. Dynamic CXR images can also capture more images within one respiratory cycle compared to expiratory and inspiratory CT images at only 2-time points. Furthermore, the registration images based on adjacent CXR images can detect pulmonary airflow within the lung field (Jiang et al., 2024), which will undoubtedly help reveal pulmonary airflow movement patterns within a respiratory cycle.

### 5.4 Limitations

Although we propose a fully automatic three-stage registration pipeline based on static P-A CXR images that can effectively register dynamic lung field images to maintain lung field morphology alignment during respiration from an engineering perspective, our research still has certain limitations. We do not have sufficient dynamic CXR images to further validate the proposed registration pipeline’s performance. Therefore, we encourage researchers to collect more dynamic CXR image images to validate the proposed registration pipeline’s performance and subsequently perform the quantitative analysis of dynamic lung fields.

## 6 Conclusion

We propose a fully automatic three-stage registration pipeline for the dynamic lung field of CXR images based on AC-RegNet with the static P-A CXR images, which effectively addresses the issue of the inability to register dynamic lung field images for the quantitative analysis of lung fields. First, the dynamic lung field mask images are generated from a pre-trained standard lung field segmentation model with the dynamic CXR images. Then, a lung field abstraction model is designed to generate the dynamic lung field images based on the dynamic lung field mask images and their corresponding CXR images. Finally, we propose a three-step registration training method to train the AC-RegNet, obtaining the registration network of the dynamic lung field images. The results show that the mean evaluation metrics of registration images based on the four basic segmentation networks with AC-RegNet achieve the mean DSC of 0.991, 0.993, 0.993, and 0.993, mean HD of 12.512, 12.813, 12.449, and 13.661, mean ASSD of 0.654, 0.550, 0.572, and 0.564, and mean MSD of 559.098, 577.797, 548.189, and 559.652, respectively. Therefore, our proposed three-stage registration pipeline has demonstrated its effectiveness in dynamic lung field registration and may become a powerful tool for dynamic lung field analysis in clinical practice.

## Data Availability

The raw data supporting the conclusions of this article will be made available by the authors, without undue reservation.
